# Long-term peripheral retinal vascular behavior in retinopathy of prematurity patients treated with ranibizumab intravitreal injection as monotherapy using fluorescein angiography

**DOI:** 10.1186/s40942-022-00402-3

**Published:** 2022-08-02

**Authors:** Raghad Al Rasheed, Mohammad Idrees Adhi, Sarah Abdullah Alowedi, Bayan Albdah, Tariq Aldebasi, Mohammad A. Hazzazi

**Affiliations:** 1 Department of Ophthalmology, King Abdulaziz Medical City, National Guard Health Affairs, PO Box 22490, Riyadh, 11426 Saudi Arabia; 2 King Saud Bin Abdulaziz University for Health Sciences, Riyadh, Saudi Arabia; 3 King Abdullah International Medical Research Center, Riyadh, Saudi Arabia

**Keywords:** Anti- VEGF, Fluorescein Angiography, Peripheral retinal vascularization, Ranibizumab, Retinopathy of Prematurity, RetCam, Reactivation of ROP, Regression of ROP

## Abstract

**Background:**

Few challenges are faced with the introduction of anti-VEGF agents as a modality of treatment for retinopathy of prematurity. The clinical behavior and time course of regression post injection differ compared to post laser ablation. This study aims to evaluate the long-term peripheral retinal vascularization outcome of Ranibizumab intravitreal injections monotherapy in the treatment of retinopathy of prematurity.

**Method:**

Hospital-based quasi-experimental study. Include ROP patients who received intravitreal ranibizumab (IVR), as primary treatment for type 1 ROP. Patients were examined under general anaesthesia to ensure documentation of all junctions of vascular and avascular zones. Images were taken by RetCam III, Phoenix ICON and fluorescein angiography was performed to describe vascular behaviors.

**Results:**

The mean gestational age was 24.67 weeks and the mean postmenstrual age at the time of intravitreal ranibizumab treatment was 36.3 weeks. Fluorescein angiography was performed at 155–288 weeks; most eyes showed two disk diameters of avascular peripheral retina. Only eyes with original aggressive ROP who required a second injection (six eyes) showed extensive peripheral avascular retina reaching zone I (13.64%). Neovascularization was evident in five eyes (11.36%), all with an original aggressive ROP and received multiple injections.

**Conclusions:**

Ranibizumab treated babies with incomplete retinal vascularization require close and long-term follow-up visits to assess post injection vascular behavior. Peripheral retinal avascular zone of more than two-disc diameters was present in most of the patients evidenced by fluorescein angiography. Babies with initial diagnosis of aggressive ROP are more likely to have persistent peripheral neovascularization.

## Background

Retinopathy of prematurity (ROP) is a complex disease process initiated in part by a lack of complete or normal retinal vascularization in premature infants. The disease can progress and contribute to blindness among these premature infants if not diagnosed and treated early. Normal retinal vascularization begins at around week 16 of gestation, starting from the optic nerve head towards the periphery. It is completed nasally at 36 weeks of gestation and temporally at 40 weeks of gestation [1]. Blair et al. aimed to quantify the amount of peripheral nonperfusion in eyes with presumed normal retinal vasculature in children; these authors defined abnormal retinal peripheral nonperfusion in children up to 13 years to be a two-disc diameter (DDs) or more of retinal nonperfusion from ora serrata [2].

ROP is classified using the International Classification of ROP (ICROP) [3], and the criteria of treatment are based on the results of the Early Treatment for ROP study (ETROP) [4]. The mainstay of treatment to the avascular ischemic retina used to be cryotherapy. Then, laser photocoagulation using diode laser was as effective as cryotherapy, with fewer treatment-related complications [4]. The BEAT-ROP study demonstrated that treatment of zone I and zone II ROP with intravitreal Bevacizumab (Monoclonal antibody, Anti VEGF) was superior to the laser treatment [5]. Moreover, phase 3 results of the RAINBOW study in 2019 presented the efficacy and safety of intravitreal Ranibizumab (IVR) (Antibody fragment, Anti VEGF) injections for the treatment of ROP [6]. In recent ICROP classification updates, regression of ROP and reactivation of ROP in relation to the post-anti-VEGF treatment of ROP have been incorporated, emphasizing their clinical importance [7].

Less myopia [8] and preservation of the peripheral visual field [9] are two proposed benefits of anti-VEGF as the main treatment for ROP. However, incomplete vascularization [10, 11], disease reactivation [12], and other vascular abnormalities, such as branching, shunt vessels, and leaking peripheral vascular abnormalities, have been reported after treatment of ROP with intravitreal Anti-VEGF [13].

Lepore et al. compared the structural outcomes of eyes treated with intravitreal bevacizumab (IVB) with fellow eyes treated with conventional laser photoablation in the zone I type 1 ROP using FA. At 4 years of age, the IVB group showed persistence of large areas of avascular retina and persistence of shunts in the periphery, suggesting continued abnormalities of circulation [13]. Meng et al. found that 36.3% of treated patients with IVR injection as monotherapy required reinjection due to recurrence. After more than 1 year of follow-up, the peripheral avascular area, which was found in 60 eyes (29.8%), was associated with lower birth weight, the severity of ROP, and repeated injections [14].

Cheng et al. described involution patterns of the vessel growth of retina of children who were treated with IVR as monotherapy for ROP up to 1 year. Thirty-four eyes of 17 children (22.1%) demonstrated finger-shaped vessels, popcorn abnormalities, arteriolar-venular shunt, and avascular peripheral retina > 2 DDs. Leakage of the neovascularization in the junction between vascular and avascular retina was found in eight eyes [15].

This study was implemented to describe the long-term vascular development of peripheral retina by use of FA in children who received IVR injections as monotherapy for type 1 ROP and to investigate the influential risk factors.

## Subjects and methods

The present study is a hospital-based quasi-experimental study performed at King Abdulaziz Medical City – Riyadh, Saudi Arabia. We carried out FA on 44 eyes of 22 patients who had earlier received intravitreal ranibizumab as monotherapy to treat type 1 ROP. Indirect ophthalmoscopy with scleral indentation with a 28 D/20 D lens was performed under general anaesthesia in the operating room. Digital retinal images were obtained before FA by a RetCam (RetCam III Imaging System Clarity Medical Systems, Pleasanton, CA, USA) and by Phoenix ICON (Phoenix ICON System Phoenix Technology Group, Pleasanton, CA, USA). The angiographic procedure included injection of 10% solution of sodium fluorescein injected intravenously at a dose of 0.1 mL/kg followed by an iso-tonic saline flush. The images were taken in the posterior pole and nasal, temporal, superior and inferior periphery in each eye, emphasizing the documentation of junctions of vascular and avascular zones. None of the patients experienced systemic complications related to FA. All photos were collected and reviewed by two vitreo-retinal consultants.

All these cases were earlier examined and treated by an experienced ophthalmologist with expertise in ROP during their stay in our hospital’s neonatal intensive care unit (NICU). The screening criteria was based on the screening guidelines laid down by the department to screen babies born on or before 32 weeks of gestation and / or birth weight of 1.5 kg or less including babies more than 32 weeks’ gestation or more than 1.5 kg birth weight whom neonatologist advised screening for associated comorbidities. The ROP screening was performed by indirect ophthalmoscopy documenting the stage, zone and presence or absence of plus disease. ROP was classified based on the ICROP revisited [3]. The number and age at which injections given were noted.

All patients had received one or more IVR; half of the adult dosage, which was 0.25 mg/0.025 mL of Ranibizumab injection as monotherapy for type 1 ROP, which included patients with aggressive ROP (A-ROP). Patients were reviewed 24 h after treatment and then examined every week until clinical evidence of regression of neovascularization and disease. We excluded patients who received other ocular interventions like laser therapy, or surgery. Also, patients with other ocular diseases like congenital cataracts, glaucoma, retinal dystrophies, or other vascular abnormalities were excluded.

The demographic information and systemic conditions were collected from patients’ charts including, perinatal data: gender, birth weight (BW), gestational age (GA), Apgar score, neonatal course, blood transfusion, and supplemental oxygen therapy.

Statistical analyses were performed using SAS for Windows software version 9.4 (SAS Institute, Cary, NC, USA). Descriptive analysis was expressed as mean ± SD for continuous variables and frequency with a percentage for categorical variables. Fishers Exact test was used for the association between categorical variables and Wilcoxon Two-sample test for continuous variables. In all analyses, a p-value less than 0.05 was considered statistically significant.

The study protocol was approved by the Research Ethics Board of the King Abdullah International Medical Research Center (KAIMRC), Riyadh, Saudi Arabia (RC19/488/R, 1-January-2020). Written informed consent was obtained from all parents who fully understood the procedure and the purpose of the study.

## Results

### Baseline and demographic data

From January 1^st^, 2016, to December 31^st^, 2018, 52 preterm infants developed type 1 ROP in one or both eyes and required treatment. Forty-four eyes (22 patients) who received IVR as monotherapy were included in this study (Table 1). Of all 22 infants, 13 were male (59%). The mean GA was 24.67 ± 1.37 weeks (median: 24, range: 23–28), and the mean BW was 707.36 ± 325.21 g (median: 625, range: 510–2100). At first IVR, the average postmenstrual age (PMA) was 36.11 ± 2.96 weeks (range: 32–46). While most eyes (77.3%) had zone II stage 3 with plus disease, eight eyes (18.8%) had A-ROP, and two eyes (4.55%) had zone I stage 3 with plus disease. Repeated IVR treatment was administered to ten eyes (22.7%) due to recurrence at PMA 42.4 ± 2.27 weeks (range: 40–45). None developed ocular or systemic complications during the follow-up period. Furthermore, none received additional laser treatment or surgical treatment.

**Table 1:** Baseline characteristics of children who were treated with IVR as monotherapy for ROP type I

Number of patients	22
Number of eyes	44
Male / Female	13 / 9
BW, mean ± SD (median, range)	707.4 ± 325.21 g (625, 510–2100)
GA, mean ± SD (median, range)	24.67 ± 1.37 weeks (24, 23–28)
PMA at treatment, mean ± SD (median, range)	36.11 ± 2.96 weeks (36, 32–46)
PMA at FA, mean ± SD (median, range)	215.32 ± 40.94 weeks (209, 155–288)

GA, Gestational age, *BW* Birth weight, *PMA* Post menstrual age, *FA* Fluorescein angiograms

### FA features

Mean adjusted age (GA and postnatal age) at FA examination was 215.32 ± 40.94 weeks (range: 155–288). The area of the avascular retina from the ora serrata was two disk diameters (2 DDs) in 36 eyes (81.8%), while the rest of them, eight eyes (18.2%), showed arrested vascularization in zone I with extensive areas of the avascular retina from the ora serrata.

### Peripheral findings

**Fig. 1:**
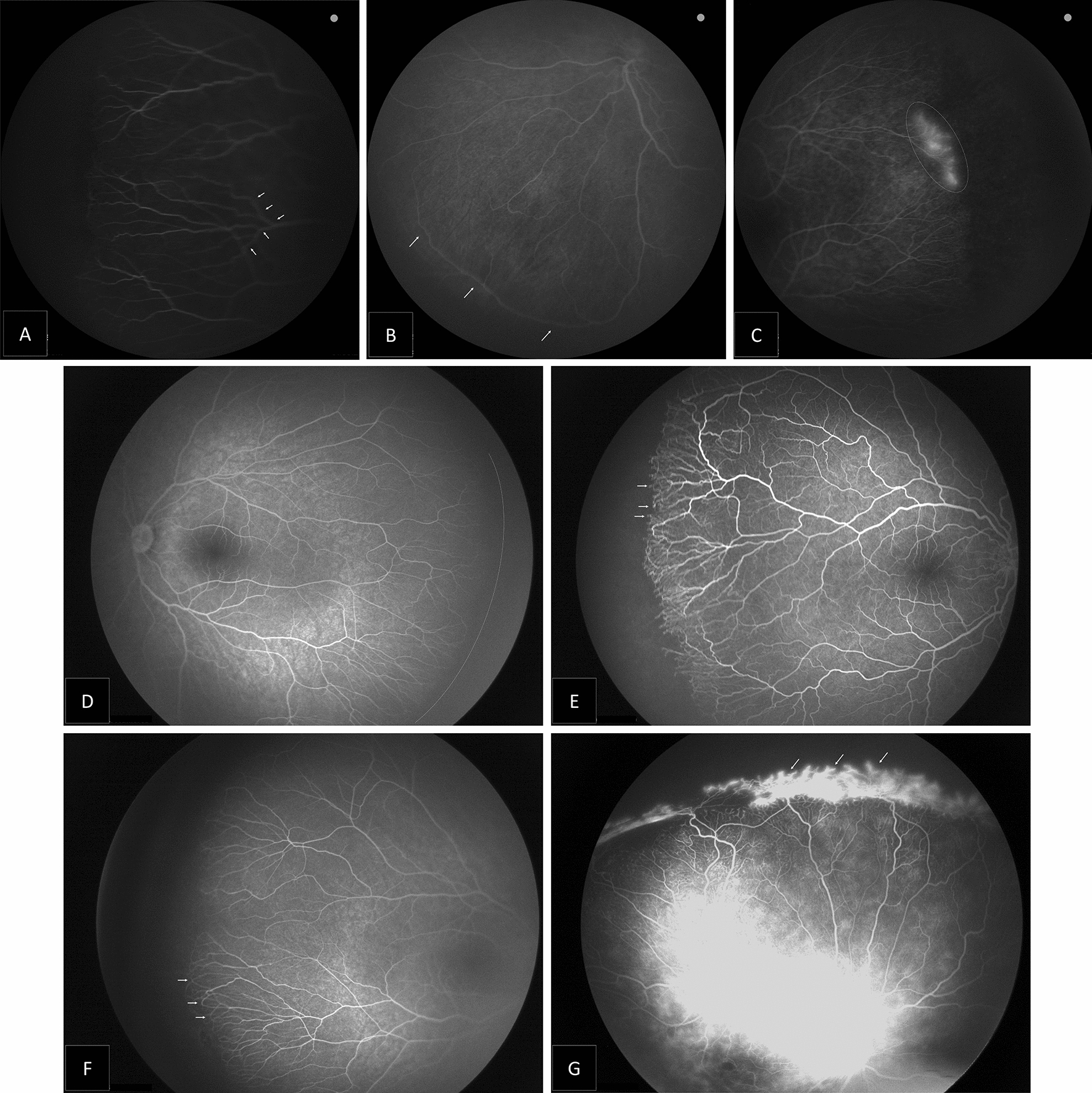
Montage of the fluorescein angiography images showing features of the junction between vascular and avascular retina in ROP patients treated with IVR: **A** Finger-like branching of vessels (arrowhead). **B** Circumferential vascular shunt at the junction (arrowhead). **C** Vascular leakage from terminal vessels at the junction area between vascular and avascular retina (dotted circle). **D** Blunted vascular terminal where the vessels ended abruptly in a stunted or blunted fashion, instead of the normal tapered endings (dotted line). **E** Popcorn vascular ending (arrowhead). **F** Finger-like anastomosis, where two adjacent terminal vessels anastomose at an apical point (arrowhead). **G** Areas of neovascularization at the junction between vascular and avascular retina (arrowhead)

These included abnormalities detected at or near the vascular-avascular junction (Table 2). An anomalous non-dichotomous, finger-like branching pattern near the junction was detected in 23 (52.27%) eyes. Arteriolar-venular shunts near the junction were found in 13 eyes (29.55%). The blunted vascular terminal was found in ten (22.73%) eyes. In six eyes, a ‘double blunted’ pattern of two circumferential rows of blunted vessel terminals was noted. Popcorn abnormalities were found only in six eyes (13.64%). Finger-like anastomosis, where two adjacent terminal vessels anastomose at an apical point, was found in 11 eyes (25%). Furthermore, there was peripheral leakage in seven eyes (15.91%); of those, five eyes (11.36%) showed formed neovascularization at the junction between avascular and vascular retina. Features of the junction between vascular and avascular retina in ROP patients treated with IVR illustrated in (Fig. 1).

**Table 2:** Fluorescein angiographic retinal vascular changes in ROP patients treated with IVR

Feature	No. of eyes (%)
**Avascular zone of peripheral retina**	
2 Disk-diameter	36 (81.8)
Posterior zone II	2 (4.55)
Zone I	6 (13.64)
**At the junction vascular–avascular retina**	
Finger-like branching	23 (52.27)
Shunts near the junction	13 (29.55)
Blunting	10 (22.73)
Double blunting	6 (13.64)
Popcorn abnormalities	6 (13.64)
Finger-like anastomosis	11 (25.00)
Leakage	7 (15.91)
Neovascularization	5 (11.36)
**Inside vascularized zone**	
Tortuosity	26 (59.09)
Focal dilation of capillaries	2 (4.55)
Loops	5 (11.36)
AV Shunt (main vessel)	8 (18.18)
Shunt crossing horizontal raphe	13 (29.55)
Capillary dropout	3 (6.82)
**Choroid and capillary bed**	
RPE atrophy	7 (15.91)
Lobular choroidal filling	15 (34.09)
Abnormal lacy or feathery retinal capillary bed	14 (31.82)
**Fovea and macula**	
Vessel encroaching onto fovea	6 (13.64)
Vessel crossing fovea	0 (0)
Absence of FAZ	0 (0)

*AV* Arterio-venous, *RPE* Retinal pigment epithelium, *FAZ* Foveal avascular zone

### Posterior pole and macular findings

**Fig. 2:**
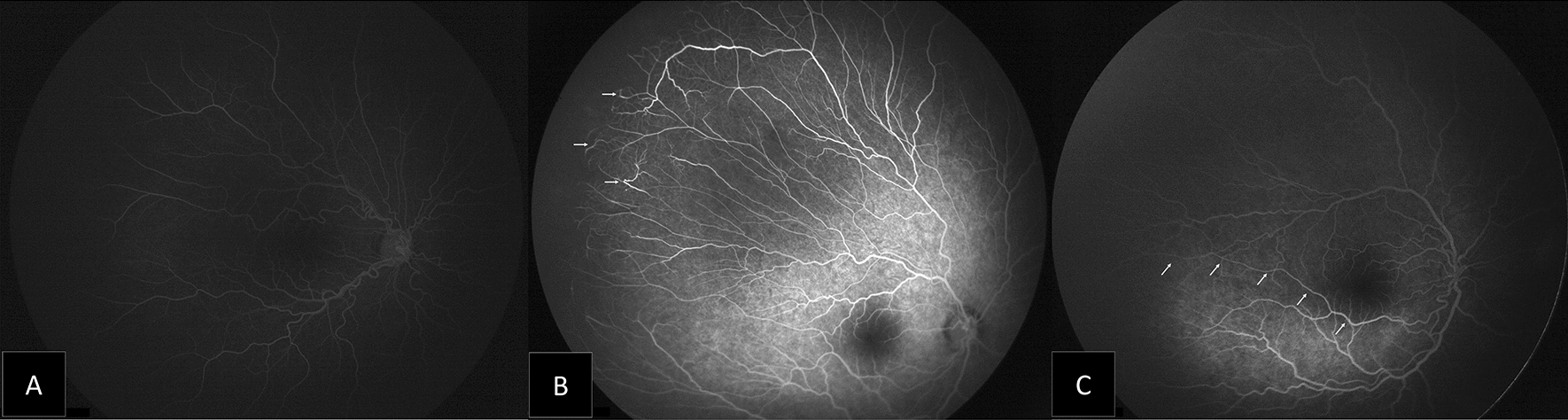
Montage of the fluorescein angiography images showing features of posterior pole in ROP patients treated with IVR: **A** Persistent vascular tortuosity. **B** Focal dilatation of capillaries (arrowhead). **C** Vascular shunt crossing the horizontal raphe (arrowhead)

**Fig. 3:**
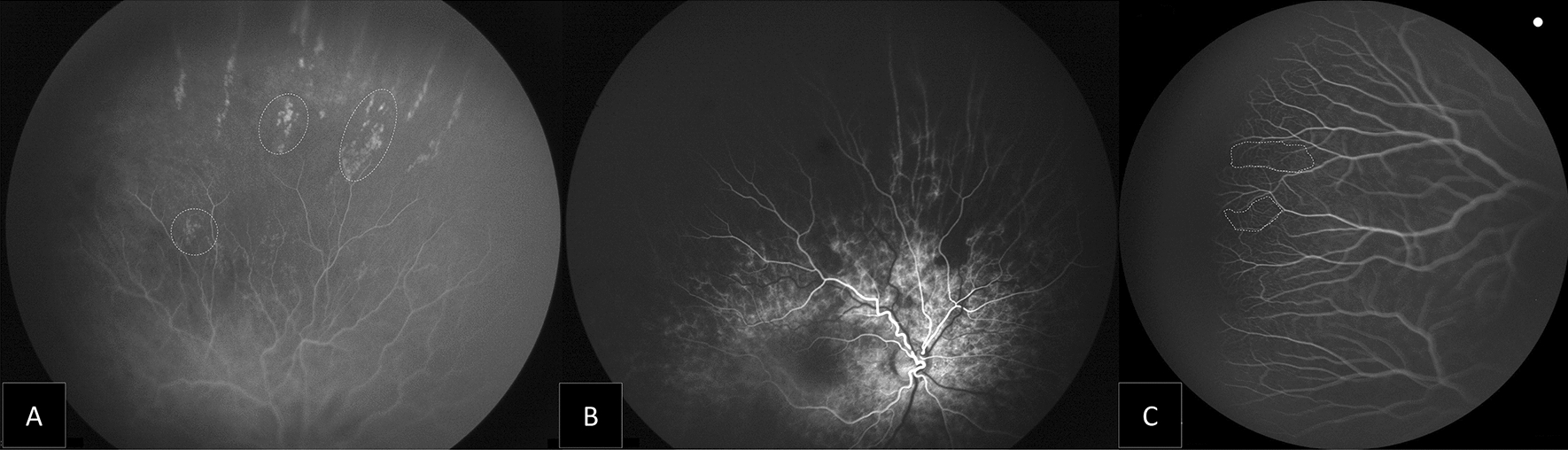
Montage of the fluorescein angiography images showing changes in choroid and capillary bed in ROP patients treated with IVR: **A** Extensive peripheral avascular area, with diffuse RPE atrophy. **B** Linear choroidal filling pattern with hypofluorescent areas. **C** Lacy or feathery retinal capillary bed (area inside dotted line)

**Fig. 4:**
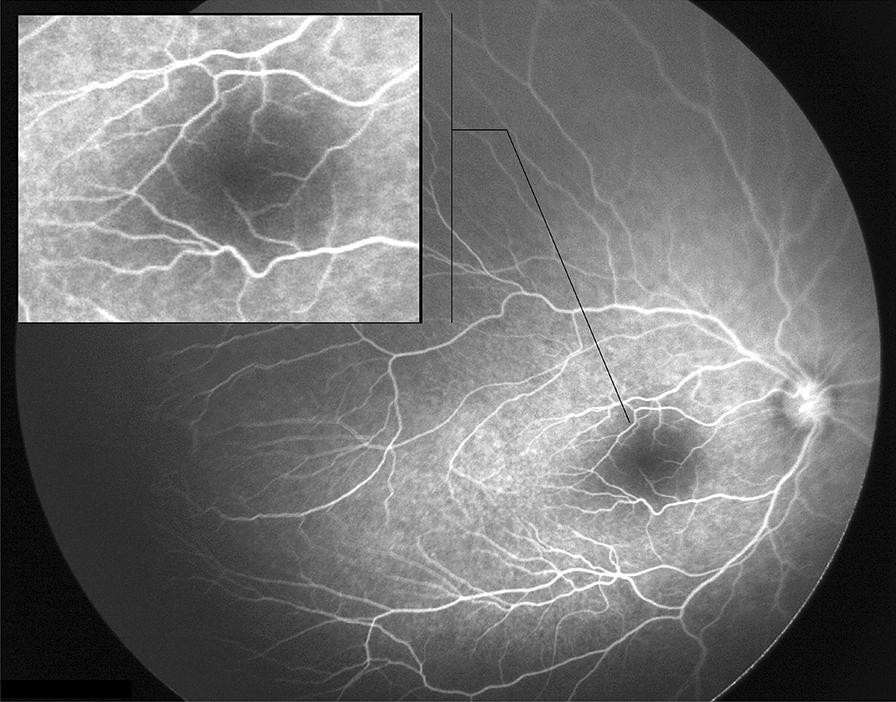
Fluorescein angiography image demonstrating changes in fovea and macula in ROP patients treated with IVR: vessels encroaching onto the fovea

Vessels were seen encroaching onto the fovea in six eyes (13.64%), no vessels were crossing the fovea. Other findings included: persistent tortuosity of vessels over the posterior pole in 26 eyes (59.09%), focal dilation of capillaries in two eyes, and loops in five eyes. Main vascular shunts were found in eight eyes (18.18%), and shunts crossing the horizontal raphe temporally were found in 13 eyes (29.55%). Areas of retinal pigment epithelium (RPE) atrophy were present in seven eyes (15.91%). Fourteen eyes (31.82%) showed retinal capillary bed changes in the form of abnormal lacy or feathery capillary bed. Choroidal filling in the lobular pattern was noted in 15 eyes (34.09%). Changes in posterior pole and macula in ROP patients treated with IVR illustrated in (Fig. 2, 3, 4).

### A-ROP

Patient characteristics and angiographic findings post IVR monotherapy for A-ROP are summarized in (Table 3). Six eyes (75%) with A-ROP required repeated treatment for disease reactivation. While only four eyes (11.8%) with original zone II stage 3 with plus disease needed a second injection. After the first treatment, repeated injections were given after 8.6 ± 1.43 weeks (median: 8, range: 7–11). Only eyes with original A-ROP and who required a second injection (six eyes) showed extensive peripheral avascular retina reaching zone I. Leakage was present in five eyes (62.6%) of A-ROP, compared to only two eyes (5.9%) with original zone II stage 3 with plus disease. Neovascularization was evident during FA examination in five eyes (62.6%). All were original A-ROP who received multiple injections.

**Table 3:** Patient characteristics and angiographic findings post intravitreal Ranibizumab monotherapy for A-ROP

No	GA	BW	PMA at FA	Eye	PMA of 1st IVR	PMA of 2nd IVR	Fluorescein Angiogram Findings		
							Avascular area at zone I (Y/N)	Leakage (Y/N)	Neovascularization (Y/N)
1	24 + 0	510	244	R	32 + 0	41 + 0	Y	Y	Y
				L	32 + 0	41 + 0	Y	Y	Y
2	23 + 0	510	205	R	34 + 0	45 + 0	Y	Y	Y
				L	34 + 0	45 + 0	Y	Y	Y
3	23 + 6	540	206	R	35 + 0	–	N	N	N
				L	35 + 0	–	N	N	N
4	23 + 4	630	209	R	34 + 0	41 + 0	Y	N	N
				L	34 + 0	41 + 0	Y	Y	Y

*GA* Gestational age, *BW* Birth weight (grams), *PMA* Post menstrual age (weeks), *R* right, *L* left, *Y*, yes, *N* no

### Multivariate regression analysis

Main risk factors: infants with a lower BW, lower GA, and receiving the first injection at a younger age were more likely to need re-treatment, to have arrested retinal vascularity at Zone I, and to develop leakage and neovascularization. Among eyes that received a second injection, treatment grade of A-ROP was significant (p = 0.0008), they were more likely to show arrested vascularity at Zone I (p < 0.0001), double blunting (p = 0.0179), leakage (p = 0.0002), and neovascularization (p = 0.0002). Eyes that showed arrested vascularity at Zone I had respiratory distress syndrome and intra-ventricular haemorrhage during their hospital course with p = 0.0159 and p = 0.0211, respectively. Also, treatment grade of A-ROP was significant (p < 0.0001), they were more likely to show leakage (p = 0.0001), and neovascularization (p < 0.0001).

## Discussion

ROP continues to be a significant cause of visual morbidity worldwide. Because of improved neonatal care in developing countries, many preterm infants survive [16]. Intravitreal anti-VEGF therapy replaces the standard laser photocoagulation for type 1 ROP. For its prompt effect, anti-VEGF can serve as monotherapy for ROP or bridge therapy until more stable conditions are assured for laser treatment. It is also more accessible and easier to administer under topical anaesthesia when compared to laser photocoagulation. However, it is crucial to be aware of the vascular changes and the behavior of treated eyes. Examination of children with indirect ophthalmoscope at pre-school age can be challenging in the clinic. Also, peripheral pathology, vascular remodeling, the boundary between the vascular and avascular retina might be difficult to appreciate using only an indirect ophthalmoscope. FA is a sensitive tool to detect vascular abnormalities in ROP compared with indirect ophthalmoscopy. With FA, we can delineate peripheral avascular area, neovascularization and leakage, and subtle capillary changes [17–19]. The use of additional modalities to describe abnormalities in eyes with ROP, such as FA and optical coherence tomography (OCT), will probably affect the traditional classification of ROP and reflect on the management and outcomes.

In this study, ten eyes (22.7%) required repeated treatment for disease reactivation; (six eyes; 75% of A-ROP, and four eyes; 11.8% with original zone II stage 3 with plus disease). Second injections were given after 8.6 ± 1.5 weeks from the first treatment. In 2018, Tong et al. studied 160 eyes with aggressive posterior ROP (AP-ROP) treated with IVR monotherapy and reported re-treatment in 82 of 160 (51%) eyes after a mean interval of 7.5 ± 6.9 weeks from the first injection [20]. Wong et al. found that reactivation after ranibizumab occurred between the 41^st^ and 42^nd^ weeks [12]. In the present study, recurrence was observed in PMA 42.2 weeks on average. Also, a group from Turkey compared the rate and interval of disease reactivation between bevacizumab and ranibizumab treatment for type 1 ROP and found a significant difference in recurrence rate between ranibizumab (50%) and bevacizumab (5.5%). Also, reactivation after ranibizumab occurred at 8.75 ± 1.5 weeks and 14 ± 2.65 weeks with bevacizumab [21]. Hoerster et al. measured serum VEGF levels in an infant treated with ranibizumab and showed that intravitreal ranibizumab reduced systemic VEGF in infants for 2–3 weeks, with a return to normal levels at 4 weeks post-injection [22]. However, intravitreal bevacizumab showed a reduction in serum VEGF levels for 2 months after injection intravitreally [23]. This highlights the importance of studying the risks and benefits of running ranibizumab for its shorter duration of systemic VEGF level suppression and the need for frequent follow-up visits for potential recurrence and re-treatment after IVR.

During our FA study, leakage was present in five eyes (62.6%) of A-ROP patients, compared to (5.9%) in eyes with original zone II stage 3 with plus disease. Also, neovascularization was evident only in five eyes (62.6%), all with an original A-ROP who received multiple injections. Mintz-Hittner et al. reported main risk factors for recurrence: low birth weight, low gestational week, and patients with disease in the zone I and diagnosed with AP-ROP [24]. Also, ROP recurrence was significantly correlated with more aggressive forms of ROP at initial treatment [25].

The extent of the vascularity into the peripheral retina is an important aspect of ROP treatment. The avascular retina normally extends ≤ 1.5 disk diameters temporally and ≤ 1.0 disk diameters nasally from the ora serrata in children up to age 13 years [2]. However, the persistent avascular retina has been reported after anti-VEGF treatment [26, 27]. In our study, all 44 eyes did not demonstrate full vascularity of less than two DDs. Also, eyes with original A-ROP and who required a second injection (six eyes) showed extensive peripheral avascular retina reaching Zone I. A retrospective study on 201 ROP-eyes treated with ranibizumab listed low BW, the severity of ROP, and repeated injections as main factors to increase the risk of the retinal peripheral avascular area [15].

Adding to the risk of an avascular retina, ROP eyes have an increased lifetime risk of lattice degeneration, retinal tears, and detachments [28]. To date, there are no clear guidelines or consensus on the management of persistent retinal avascularity after anti-VEGF treatment for ROP and no clinical trials on the efficacy and safety of laser in this context. Some elected to apply prophylactic laser for persistent avascular retina in ROP eyes post intravitreal injection of anti-VEGF agents [29]. We recommend laser ablation of the ischaemic peripheral retina in the eyes, which showed persistent neovascularization at the vascular and avascular retina junction on FA. A comprehensive discussion on the pathology and possible treatment options with the family is essential. Potential options include prophylactic laser photocoagulation to areas of retinal nonperfusion. Another option is long and close observation with FA study to delay laser ablation for worsening leakage.

The small sample size limited this study’s conclusions. The data collected during patients’ stay in NICU are retrospective. The sample size was insufficient to comment on disparities between disease categories; all patients with type 1 ROP, including those with A-ROP, had similar FA changes. Age at the time of FA was variable among study participants, which allows us to conclude that vascular abnormalities can occur at any age after ranibizumab treatment, but data is not sufficient to comment on age-specific vascular abnormalities. Prospective studies with larger sample sizes must set treatment paradigms and monitoring protocols.

## Conclusions

Ranibizumab monotherapy showed good results in treatment of ROP Type I. However, FA results of 22 children at 288 weeks PMA after primary treatment of ROP Type I with Ranibizumab revealed the presence of peripheral retinal avascular zone of more than two-disc diameters in most of the patients. Babies with initial diagnosis of A-ROP are more likely to have persistent peripheral neovascularization at the junction of vascular and avascular areas which may extend posteriorly into zone I. Persistent avascular zones with neovascularization should be treated with peripheral laser ablation. In the absence of clear guidelines for management of babies with persistent avascular zone without neovascularization, if long-term follow up unwarranted, prophylactic peripheral laser ablation is highly recommended.

## Data Availability

The datasets used and/or analyzed during the current study are available from the corresponding author on request.

## Abbreviations

A-ROP: Aggressive retinopathy of prematurity
BW: Birth weight
DD: Disc diameter
ETROP: Early treatment for ROP study
FA: Fluorescein angiography
GA: Gestational age
ICROP: International classification of ROP
IVB: Intravitreal injection of bevacizumab
IVR: Intravitreal injection of ranibizumab
NICU: Neonatal intensive care unit
OCT: Optical coherence tomography
PMA: Post-menstrual age
ROP: Retinopathy of prematurity
RPE: Retinal pigment epithelium
VEGF: Vascular endothelial growth factor
